# The Minocycline Ameliorated the Synaptic Plasticity Impairment in Vascular Dementia

**DOI:** 10.22037/IJPR.2020.113942.14576

**Published:** 2021

**Authors:** Mohammad Davood Sharifi, Narges Karimi, Mohammad Karami, Afshin Borhani Haghighi, Mohammad Shabani, Mahnaz Bayat

**Affiliations:** a *Imam Reza Hospital, Faculty of Medicine, Mashhad University of Medical Sciences, Mashhad, Iran. *; b *Department of Physiology, The Medical School, Shiraz University of Medical Sciences, Shiraz, Iran. *; c *Clinical Neurology Research Centre, Shiraz University of Medical Sciences, Shiraz, Iran. *; d *Neuroscience Research Center, Neuropharmacology Institute, Kerman University of Medical Sciences, Kerman, Iran.*

**Keywords:** Chronic cerebral hypoperfusion, Bilateral carotid occlusion, Minocycline, Synaptic plasticity, Neurotransmitter release probability, Basal synaptic transmission

## Abstract

Chronic cerebral hypoperfusion (CCH) leads to vascular dementia with progressive hippocampal damage and cognitive impairments. In the present *study*, we compared early and late Minocycline (MINO) treatment on cognitive function, long and short-term synaptic-plasticity following CCH. We used bilateral common carotid arteries occlusion model (2VO) for induction of hypoperfusion. Male Sprague-Dawley rats were divided into 5 following groups (each having 2 subgroups): 2VO + V (vehicle), 2VO+MINO-E (early treatment of MINO on days 0 to 3 after 2VO), 2VO+MINO-L (late-treatment on days 21 to 32 after 2VO), control, and sham. Passive-avoidance (PA) and radial arm maze (RAM) tests were used to investigate learning and memory. Long term and short term synaptic plasticity were assessed by field potential recording, the brains were removed after recording and preserved for histological study to count pyramidal cells in CA1 region.Cerebral hypoperfusion could impair memory performance, synaptic plasticity, and basal synaptic transmission (BST) along with hippocampal cell loss. Thus, we found a significant reduction in step-through latency (STL) of PA test with a higher number of working and reference errors in RAM in CCH rats. However, only late treatment with MINO improved memory performance, synaptic plasticity, hippocampal cell loss, and increased neurotransmitter pool (NP) in CCH rats, but early treatment could not produce long-lasting beneficial effects 32 days after 2VO. MINO may improve synaptic plasticity and memory performance in hypo-perfused rats directly and indirectly by increasing NP and/or suppressing inflammatory factors, respectively.

## Introduction

Brain has a high demand for blood and energy, and it is so susceptible to the impacts of perfusion impairments. Such impairments can start with a sudden stop in blood flow to the brain as it frequently happens during strokes or it can progress gradually by vascular dysfunctions that may lead to chronic cerebral hypoperfusion (CCH) during life (1). Among such conditions, CCH is considered an crucial etiological factor in the progression of vascular dementia and Alzheimer’s disease AD (2). The hippocampus and cerebral cortex are the most vulnerable areas to the impacts of hypoperfusion, which are critically involved in cognitive and memory functions (3). It is well known that long-term potentiation (LTP) in the hippocampus is the cellular basis for memory formation (4), and much of the relevant experimental work on neurodegenerative disorders used synaptic plasticity as a good index for evaluation of learning and memory (5, 6). Although the cause of memory and cognitive deficit is still not exactly known in CCH, existing research recognizes a critical role of inflammation (7) Therefore, available anti-inflammatory drugs have been suggested as a therapeutic approach (7).

It has been suggested that the Minocycline (MINO), a second-generation and semi-synthetic of tetracycline, has antioxidant, anti-inflammatory and direct neuroprotective properties along with antibiotic effects (8). The pleiotropic actions of MINO include increasing effects on brain-derived neurotrophic factor (BDNF) levels, anti-apoptosis activities (9), inhibition of metalloproteinase, protection of blood-brain barrier (10), and lipophilic properties with high permeability of the blood-brain barrier (BBB), led to a significant area of interest as a novel viable treatment for focal and chronic cerebral ischemia, inflammatory disorders such as multiple sclerosis, hepatic encephalopathy, rheumatoid arthritis and even in psychiatric disorders (11-13). Data from several studies suggest that MINO has neuroprotective effects in AD (14), Parkinson’s disease (15), Hepatic encephalopathy (12), and spinal cord injury (16). It is thought that these actions are distinct from its antibiotic abilities and could be due to the direct neuroprotective effects of the drug. Notably, it has been shown that MINO restores learning and memory deficit in aged mice via enhancing synaptic plasticity and synaptogenesis (17). So far, however, there has been little discussion about MINO effects on cognitive impairment following vascular dementia (18-21), and most studies in the field of CCH have only focused on molecular and behavioral effects of MINO. Thus, previous studies have not dealt with the evaluation of electrophysiological properties of MINO in CCH. Such approaches, however, have failed to address the effectively neuroprotective action of MINO, especially on synaptic plasticity, learning and memory. Therefore, this study set out to investigate the usefulness of MINO neuroprotective effects on hippocampal synaptic plasticity, learning, and memory. In addition, about the best starting time of treatment and long lasting effects with MINO in CCH there are controversy. Thus, with administration of MINO at the early and late stages of CCH, we evaluated the long lasting beneficial effects of early treatment with MINO on synaptic plasticity.

## Experimental

*Animals and grouping*

Adult male Sprague Dawley rats (bodyweight 220-250 g, about 6 weeks of age) were housed in a 12:12 h light: dark cycle with free access to food and water in a temperature and humidity controlled facility. 7 days before surgery, the animals were adapted to new conditions with daily handling. The animals were randomly divided into 5 groups, each having 2 subgroups, including the control group (n = 18, 9 rats in each subgroup), sham group (n = 16, 8 rats in each subgroup), 2VO +V (vehicle) group (n = 18, 9 rats in each subgroup), 2VO+ MINO-E group (n = 20, 10 rats in each subgroup), and 2VO + MINO-L group (n = 20, 10 rats in each subgroup). The first subgroup was used for passive avoidance test and field potential recording, and at the end of the recording, the animal’s brains were removed for histological experiments. The second subgroup was used for the radial arm maze (RAM) test.

Minocycline hydrochloride (50 mg/Kg, sigma) was prepared in filtered PBS and pH adjusted in7.4. The animals received the first dose of intraperitoneal (i.p) at day 0 immediately after the surgery until day 3 in 2VO +MINO-E group, but in 2VO + MINO-L group, i.p injection of MINO started at day 21 after the surgery and continued until day 32.

*Surgical procedure*

All the animal care and surgeries were performed according to the animal care and ethics guideline of Shiraz University of Medical Sciences, and all effort was made to minimize the pain and discomfort in animals. CCH was induced using the irreversible two-vessel occlusion model according to our previous study (22). Briefly, after 12 h of fasting and 4 h of thirst, rats were anesthetized with Ketamine (80 mg/kg) and Xylazine (10 mg/kg) and placed on the heating blanket in the supine position after shaving and disinfecting the ventral neck area the small incision in midline of the neck was made. Surgery was performed under the surgical microscope magnification. The angle between the sternohyoid and sternocleidomastoid muscles was bluntly dissected very carefully to reach common carotid arteries; then, by very thin dissection, the adventitial sheaths were freed, and the branches of vagal as well as sympathetic nerve were separated. Common carotid arteries bilaterally ligated permanently without any vessel damage with 4-0 silk suture. The incision was sutured, and the animals were kept at 37 ºC temperature until full consciousness as during the whole surgery. The sham-operated rats received all the surgical procedures without ligation of vessels. 

*Behavioral experiments*

*Passive avoidance test (PA)*

A passive avoidance test was performed to evaluate learning and memory by a shuttle box apparatus as explained in previous studies (23, 24). Briefly, the box has two light and dark rooms connected by a guillotine door. For adaptation, on the 31^th ^day after the vessel occlusion, each rat was placed in the light room and allowed to step into the dark room, but for the learning trial, the animal was punished with an electrical shock (0.5 mA, 50 Hz, 2 s once) upon entrance to the dark room. For all animals, the learning procedure is repeated every five minutes until they learn the task. For evaluation of retention, 24 h after the learning trial, the test was performed by placing each animal in the light room. The time course that each animal spent in the light room before entering the dark room was recorded as step-through latency (STL). The maximum cutoff time for the STL was 300s without foot shock. 

*Radial arm maze test (RAM)*

For evaluating spatial learning and memory, we used the RAM test conducted in 3 phases (shaping, acquisition, and retention). More details about this test are explained in our previous work (22). The number of reference memory errors, working memory errors blindly recorded. 25 days after surgery, acquisition trials started (4-5 trials per day) until they reached criterion (one error or no errors during the 3 consecutive trials) or a maximum of 20 trials. 4 days after the final acquisition trials, retention testing was performed. 

*Field potential recording*

Thirty-two days after induction of hypoperfusion field potential recording (Electromodule-R12, Science Beam, and Tehran, Iran) was performed with our previously published protocol (23). Rats were anesthetized with urethane (1.5 g kg-1) and fixed on a stereotaxic apparatus. The skull was exposed, and two holes were drilled according to the positions obtained from Atlas of Paxinos, one for recording in CA1 region (-3AP and 2L) and another for stimulation of Shaffer collateral pathway (-4AP and 3L). The bipolar stainless steel electrodes (0.2 mm diameter, Advent, UK) were lowered elegantly to obtain the best possible hippocampal pulse. Monophasic square waves were used for stimulation. The electrical signals amplified 1000 folds, digitized at 10 kHz, and filtered at 1-3 kHz (25, 26). 

Input/output recording started after 30 minutes’ stabilization by a series of stimulation impulses from the lowest strength until the maximal fEPSP amplitude was obtained. A 20-minute basal recording and paired-pulse recordings were done before induction of High-frequency stimulation (HFS). The paired-pulse ratio (PPR) was used to determine short-term synaptic plasticity and release probability. The PP index was as the ratio of the second response amplitude to the first one (pulse2/pulse1) With inter-stimulus intervals of 20, 25, 50, 100, 150, 200, and 250 ms. The HFS was delivered for LTP (long-term potentiation) induction with 3 × 10 trains of 20 pulses at 200 Hz. The fEPSP recording was continued for 60 min after HFS. A second round of paired-pulse recording was also run at the end of the experiment. 

*Histological study*

The brains were fixed through transcardial perfusion with paraformaldehyde solution at the end of the field potential recording. Each brain was kept in 4% buffered formalin solution and used for 10 μm coronal serial sectioning through the CA1 region of the Hippocampus. To increase the differentiation of nuclei to background staining, 0.5% acetic acid solution was used, and then deparaffinization and hydration were performed. Paraffin-embedded slides were stained using Giemsa solution (Merck, Germany) for 45 min. In CA1 area, three sections were selected with equal intervals, and from each, 10 fields were selected randomly (each field with the area of 1369 μm^2 ^and magnification of × 600) cells with intact, bright, and round nuclei were counted. Pyknotic cells with deformed and condensed nuclei were counted again from 10 fields with the area of 42870 μm^2 ^(×100 magnifications) in each of the three sections.

*Statistical analysis*

The data are presented as mean ± SE. One-way ANOVA was used for comparison of PA, RAM results, maximum stimulation response, paired-pulse ratio, and the number of neurons between the 5 groups. We used two- way ANOVA to evaluate fEPSP amplitude in the I/O curve and two-way repeated-measures ANOVA to evaluate the time and interaction (time and group) effects for fEPSP changes after delivery of HFS. The amplitude of fEPSP was normalized and the paired t-test was used to compare the PPR before (baseline) and after tetanus stimulation. For all data, the differences were considered significant at the level of *P* < 0.05.

## Results

In this study, all obtained results from the sham group were identical to the control group, and no significant differences were found between sham and control groups. Thus, we reported only the control group in the presentation of the results. 

*Behavioral results*

*Late treatment of Minocycline improved memory deficit in hypo-perfused rats*

**Figure 1:**
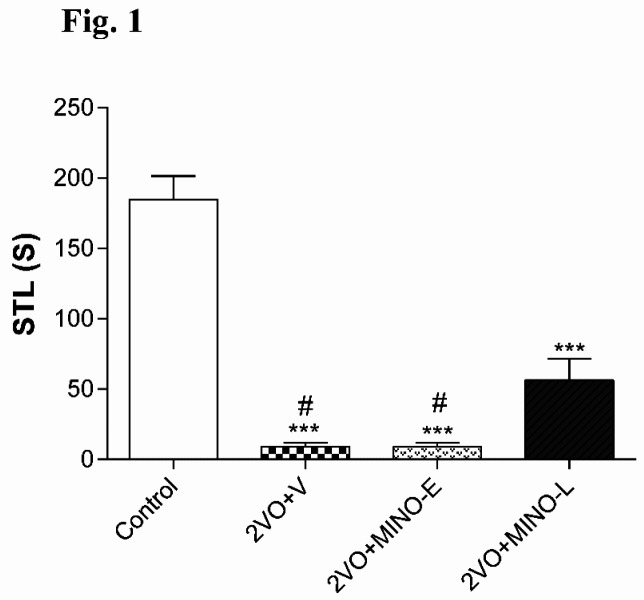
The administration of MINO at the late stage, partially improved STL time in hypo-perfused rats. The STL time in passive avoidance test, the values are shown as mean ± SEM . Significant differences with respect to the control (^***^P < 0.001) and 2VO+MINO-L (^#^*P* < 0.05) groups

The STL time of 2VO+V (9 ± 3.02s) and 2VO+MINO-E (9 ± 2.73 s) groups were significantly (both < 0.001) shorter than control animals (184.7 ± 16.61s). However, the administration of MINO at the late stage partially increased the STL time to the level 56.2 ± 15.2 s, which was still lowered with respect to the control group, but significantly higher (both; *P *< 0.05) compared to values of 2VO+V and 2VO+MINO-E groups (Figure 1).

**Figure 2:**
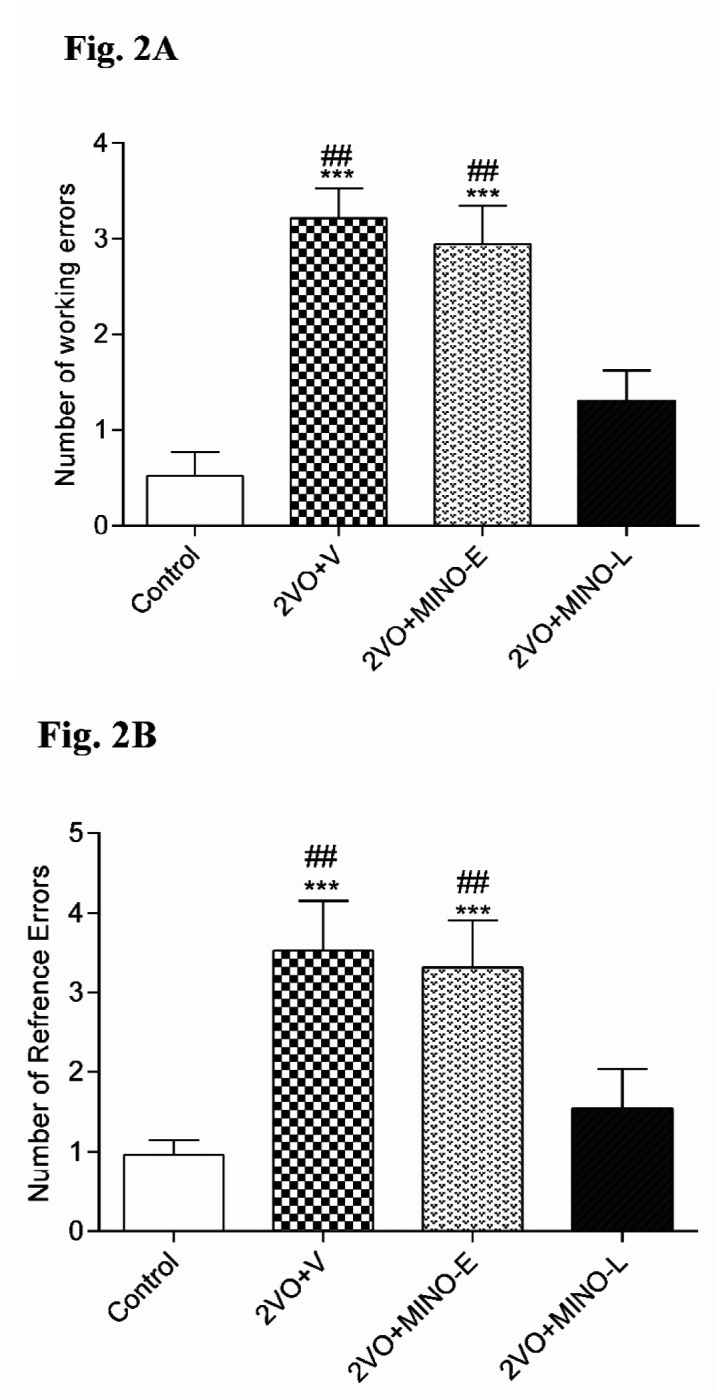
The administration of MINO at the late stage, improved spatial memory in hypoperfused rats. (A) The number of working errors, (B) reference errors. Values are shown as means ± SEM. ^*** ^(*P* < 0.001); and ^## ^(*P *< 0.01) represent the significant difference *vs. *control and 2VO + MINO-L respectively

In addition, the obtained results from RAM test are presented in Figure 2 showed that hypoperfusion significantly (*P < *0.001) increased the working (3.21 ± 0.316), reference errors (3.52 ± 0.631), and the number of trials (18 ± 2.74) than the control group. However, the levels of working (2.94 ± 0.403) and reference errors (3.31 ± 0.596) in 2VO+MINO-Egroup were still higher with respect to the control group, but 2VO+MINO-L group exhibited a significantly (*P < *0.01) lower number of working (1.305 ± 0.318) and reference errors (1.54 ± 0.493) respect to the both 2VO+V and 2VO+MINO-E groups, the levels of errors in this group compared with the control group. Therefore, only late treatment with MINO ameliorated radial maze memory impairment induced by the 2VO model (Figs. 2A and B). MINO could not recover learning performance (trails number) in hypoperfused rats.

*Electrophysiological results*

*Minocycline could not improve the depressed basal synaptic transmission (BST) of CA1 neurons in hypo-perfused rats*

The BST of CA1 neurons were examined by Input-Output (I-O) with stimulation intensities (SI) ranging from 50 to 1200 μA in 50 μA increments.

**Figure 3:**
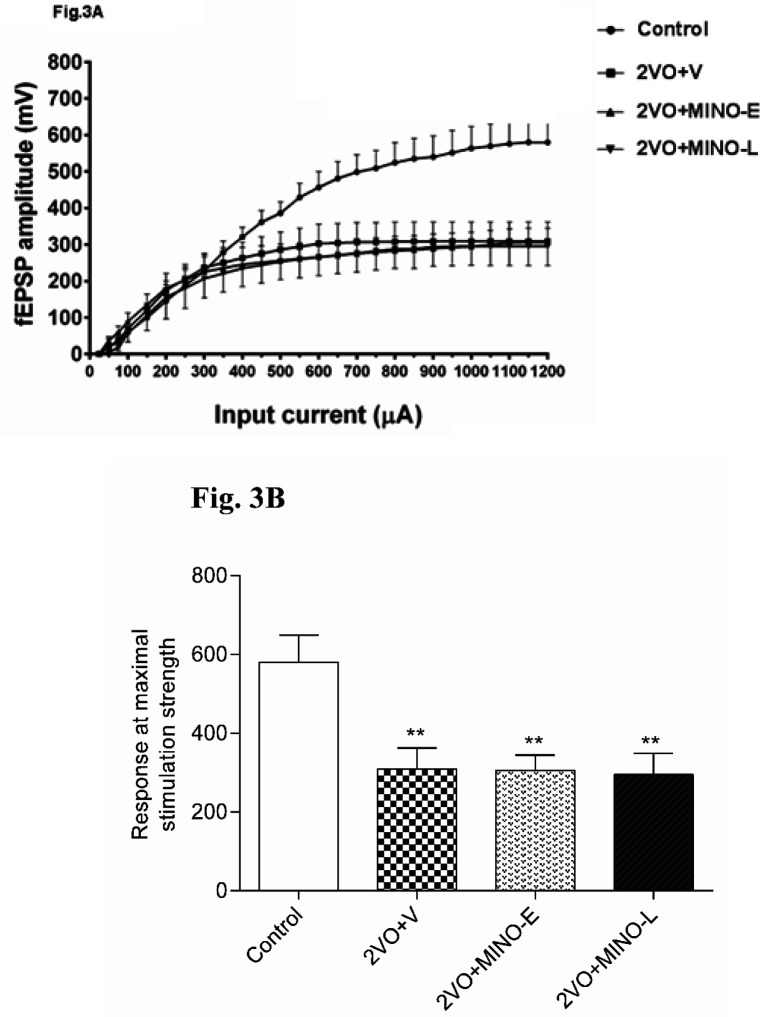
The CCH strongly decreased the BST of CA1 neurons and administration of MINO failed to recover this effect. (A) input/output (I/O) curve and (B) maximum stimulation strength response. Values are expressed as mean ± SEM. ^**^P < 0.01 versus control group

Figure 3A shows that the I/O curves were shifted downward in 2VO and all treated groups compared to the control group. This means that cerebral hypoperfusion markedly depressed the BST of the CA1 neurons, and MINO could not improve the BST of CA1 neurons in the 2VO model.

The maximal fEPSP amplitude was evaluated in more detail, and Figure 3B shows that response of 2VO group significantly (*P < *0.01) was lower (309.2 ± 53.01 mV) in comparison to the control group (592 ± 76.36 mV). However, in the 2VO+MINO-E and 2VO+MINO-Lgroups, the responses were also lowered (305.9 ± 38.7 mV 295.3 ± 53.9 Mv respectively), and both groups showed significant differences (*P < *0.01) compared to the control group (Figure 3B). In addition, no significant difference between the two treated groups was evident.

*Late treatment of Minocycline increased the robustly short-term plasticity in hypo-perfused rats*

**Figure 4:**
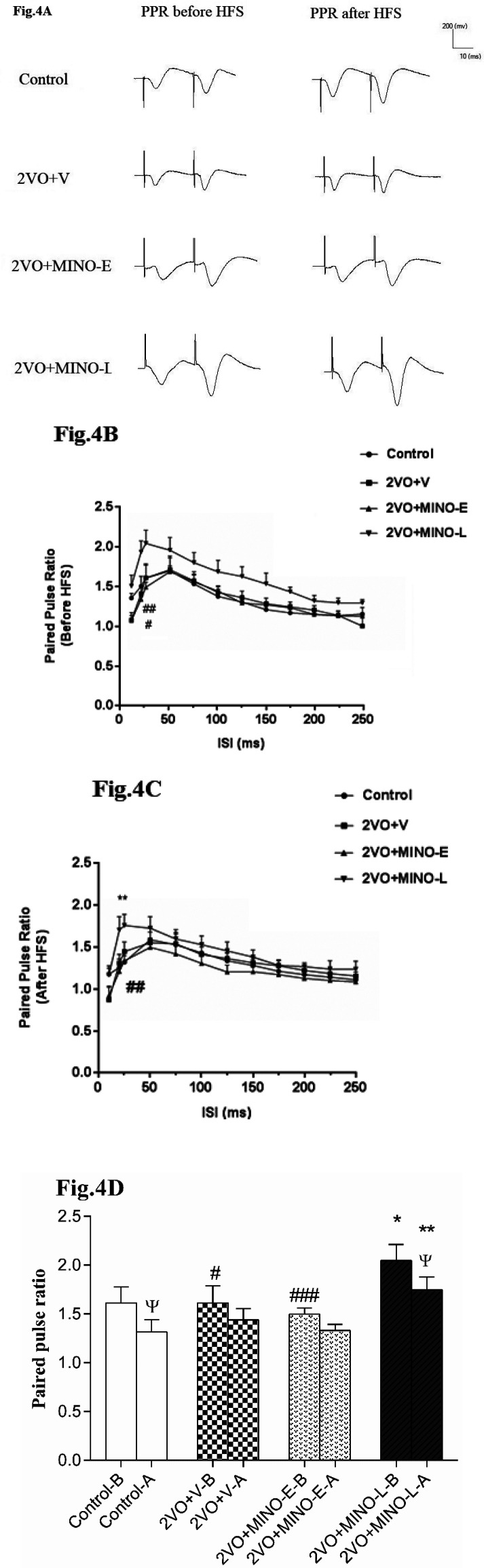
The effect of MINO administration on the PPR (fEPSP2/fEPSP1) in hypoperfused rats. (A)The sample traces are presented for ISI 25 ms in before and after HFS stimulation. (B and C) The linear graph shows the PPR change at ISI 20–250 ms before and after HFS. (D)The Paired-T test comparison of PPR before and after HFS for ISI 25 ms. Values shown as means ± SEM, significant differences with respect to the control (^*^P < 0.05, ^**^P < 0.01) and 2VO+MINO-L (^#^*P* < 0.05, ^##^*P* < 0.01 and ^###^*P* < 0.001) groups. Significant difference before and after HFS ^Ψ^*P* < 0.05

Calculation of paired-pulse ratio PPR (fEPSP amplitude P2/P1) was used to study short-term synaptic plasticity. From the data in Figures 4A and B, it is apparent that the levels of PPR for all ISIs before and after HFS in 2VO+V group are comparable with respect to the control group (Figure 4). However, the most interesting aspect of these graphs is that the late treatment with MINO significantly increased PPR before and after HFS with respect to values of control and 2VO+MINO-E groups. In addition, it is apparent that the level of PPR for ISI 25ms in the control group was depressed by delivery of HFS from (1.61 ± 0.16) to (1.31 ± 0.12), but the 2VO+V (1.61 ± 0.17 *vs.* 1.44 ± 0.11) and 2VO+MINO-E (1.49 ± 0.06 *vs.* 1.33 ± 0.06) groups did not show this depression (Figure 4D). However, treatment with MINO in the late stage of 2VO led to a significant decrease of PPR from 2.04 ± 0.16 to 1.74 ± 0.13 (*P < *0.05). 

*Late treated of Minocycline improved long-term synaptic plasticity impairment at Schaffer collateral-CA1 synapse in hypoperfused rats*

**Figure 5:**
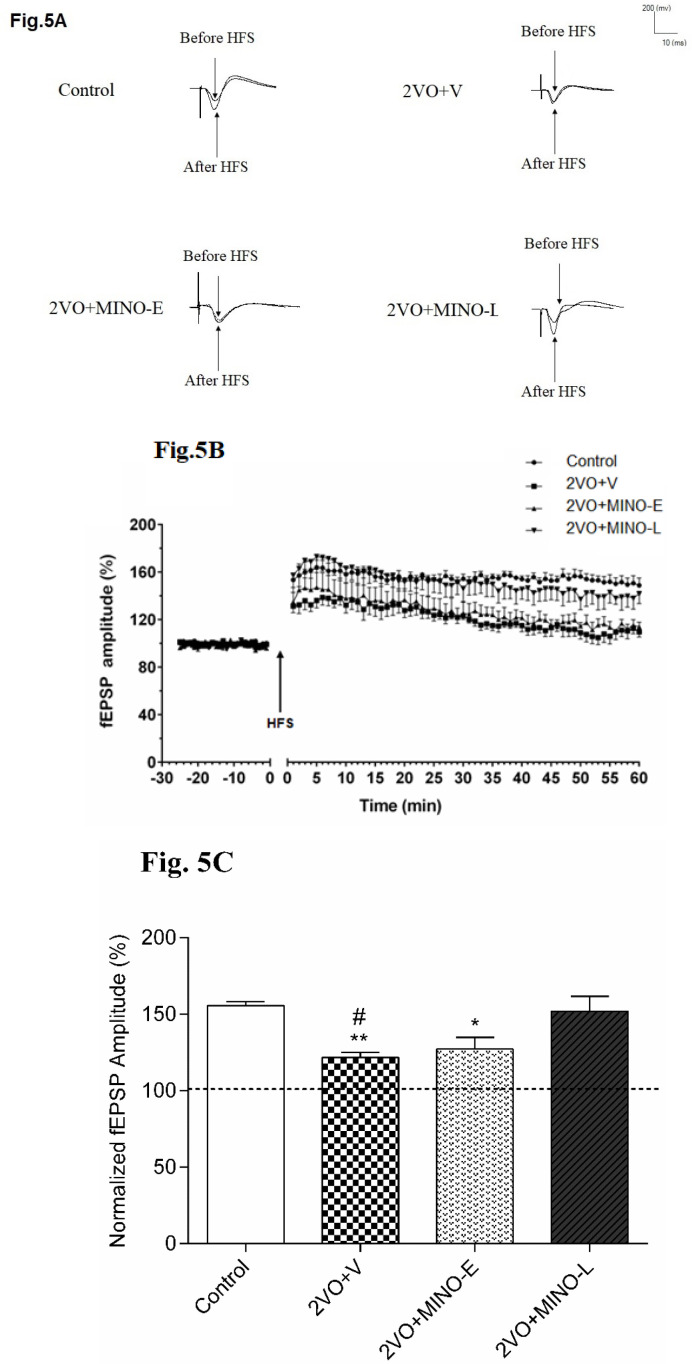
The MINO administration improved long-term synaptic plasticity (LTP) in hypoperfused rats. (A)The sample traces of responses and level of LTP induction. (B)The values are expressed as percent change of fEPSP amplitude relative to the baseline after HFS stimulation; the upward arrow indicates HFS delivery. (C)The mean of 60-minute percentage change of fEPSP slope after HFS in each group. Values are shown as means ± SEM, significant differences with respect to the control (^*^P < 0.05 and ^**^P < 0.01) and 2VO+MINO-L (^#^*P* < 0.05) groups

It is obvious from the sample traces in Figure 5A and plots of normalized fEPSP amplitudes in Figure 5B that LTP was induced by the HFS delivery to the Schaffer collateral-CA1 synapses in control, and 2VO+MINO-L groups, but not in the 2VO+V and 2VO+MINO-E groups. In addition, the mean alterations of fEPSP amplitude (percentage from baseline) for 60 min after delivery of HFS was calculated (Figure 5C) in control (155.5 ± 2.06%) and 2VO+MINO-L (151.9 ± 9.7%) with no statistical differences.

In contrast, the impairments of LTP induction in the 2VO+V and 2VO+MINO-E groups were shown with the low and comparable values for mean of percentage changes of fEPSP after HSF ( 121.6 ± 3.29% and 127 ± 7.7%) respectively, with significant differences versus the control and 2VO+MINO-L groups, (*P* < 0.05 and *P* < 0.01) and (*P* < 0.05). 

*Stereological results*

*Late treated of Minocycline improved cell survival and decreased Pyknotic cells in hypoperfused rats*

**Figure 6:**
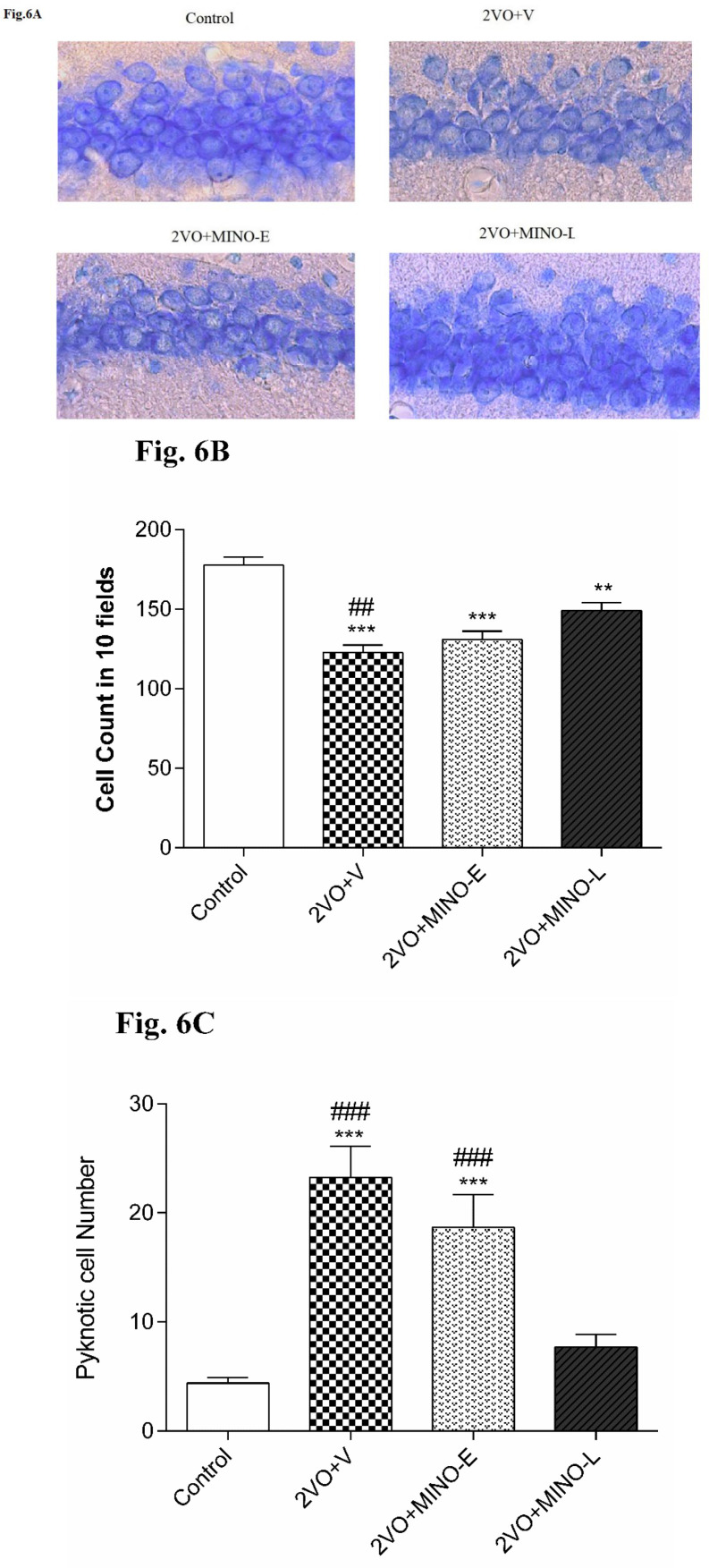
Late treatment with MINO increased cell survival of CA1 pyramidal neurons and decrease Pyknotic cell number in hippocampus. (A-C) The representative light microphotographs for CA1 pyramidal neurons, total pyramidal cell number, and Pyknotic cells number. Values are expressed as mean ± SEM. Significant differences with respect to the control (^**^P < 0.01 and^***^P < 0.001) and 2VO+MINO-L (^##^*P* < 0.01 and ^###^*P < *0.001) groups

Counting of intact pyramidal cells in 10 fields of 1369 μm^2^area showed a significant decrease (*P < *0.001) in 2VO+V group (122.7 ± 5.03) compared to Control (177.6 ± 4.94) Figure 6A. Minocycline treatment for 4 days did not change the loss statistically but 12 days of treatment had significantly (*P < *0.01) restored the pyramidal cell counts (149.1 ± 5.03) still it was significantly lower that the intact animals (*P < *0.01). To have a more accurate judgment, more significant fields (42870 μm^2^) were adjusted for counting pyknotic cells Figure 6B. Accordingly, in the two groups of 2VO+V and 2VO + MINO-E, much more (*P < *0.001) pyknotic pyramidal cells were counted (23.2 ± 2.8 and 18.6 ± 3 resp. *vs.* 4.4 ± 0.5 in the control group). Pyknotic cells were significantly (*P < *0.001) lower in 2VO+MIN-L (7.7 ± 1.1) compared to 2VO+V and 2VO + MINO-E.

## Discussion

The present study was designed to compare the possible beneficial effect of MINO at the early and late stages of vascular dementia on hippocampal synaptic plasticity and cognitive impairment. MINO is used as an antibiotic in the clinic, and it can easily cross the blood-brain barrier. As mentioned in the literature review, it acts as an antioxidant, anti-inflammatory, and anti-apoptotic factor in neurodegenerative disorders (27). Although inflammation has shown an essential role in memory deficit following dementia. However, contradictory reports still exist and as far as we know, the exact mechanisms and long lasting beneficial effects of MINO on memory and hippocampal synaptic plasticity in 2VO model have not been completely understood. 

Consistent with the literature, we found that 2VO impairs hippocampal synaptic plasticity and memory performance (22, 28). However, administration of MINO at days 21-32 after 2VO induction improved memory performance, long and short term synaptic plasticity as well as cell survival, but early treatment (days 0-3) was failed to recover these parameters and did not have any long term beneficial effects in the chronic phase of VD. Nevertheless, treatment with MINO in both timelines was failed to improve the BST. While this result is contrary to several reports that showed long-lasting beneficial effects of different anti-inflammation agents in the acute phase of 2VO. Ma, *et al.* (2015) found that the MINO treatment at the early stage (days 0-3), similar to late-stage (days 4-32) after unilateral carotid occlusion produced long-lasting effects (20). Thus, we hypothesized that MINO administration in the first 3 days after 2VO may have long-lasting beneficial effects in the chronic phase on memory and synaptic plasticity impairment but we found that early administration of MINO could not produce long lasting improvements after 2VO. Although, in early group the assessment of different parameters in our study was performed far long after early minocycline administration, the parameters were evaluated at the same day after surgery in both group. We should consider that the time is important factor for evaluation of symptoms in vascular dementia. Because it is well known that symptoms gradually worsen over time and the animals need at least 4 weeks to develop dementia symptoms, we need to evaluate early and late group at same time after induction of VD. Yue, *et al*. (2018) also published a paper that showed the deactivation of the HMGB1, a pro-inflammatory molecule, in the acute phase of ischemia can lead to suppression of hippocampal inflammatory responses and showed a long-lasting beneficial effects after 2VO (29). It is encouraging to compare these data with those found by Sa Santos* et al. *(2016) who reported that treatment with Ibuprofen-conjugated kyotorphinsat at days 7-14 after 2VO improved cognitive/behavioral functions and hippocampal pathology at week 6 post-surgery (30). However, the result of our study suggested that four doses of MINO administration in acute phase (day 0-3) could not exert long lasting beneficial effects in chronic phase. This discrepancy could be attributed to the different mechanisms involved in the anti-inflammatory action of the drugs and may be due to the different methods used for induction of chronic cerebral hypoperfusion or evaluation of memory performance. It seems possible that, other factors more potent than inflammation following 2VO responsible for cognitive impairment or different pattern of pro-inflammatory secretion in early and late stages of 2VO caused this controversy. It is now well established that high expression levels of the pro-inflammatory cytokine-tumor necrosis factor alpha (TNF-α) and interleukins (IL-1β&IL-6) leads to neuro inflammation (29, 31, 32) in acute (3 days) as well as chronic phases (4 w and 12 w) of 2VO model (29). These factors may explain the relatively good long-term NSAIDs-based therapies and cognitive improvement in dementia (33, 34). Therefore, in our study the doses of MINO administration in acute phase needs to be repeated or if we had another treatment group that received MINO at day 4-32 after 2VO surgery it was likely to show more potent anti -inflammation effect in synaptic plasticity and cognitive improvement than MINO administration at day 21-32.

Next, we evaluated the electrophysiological properties of 2VO rats in response to MINO in more mechanistic detail. In addition, we found a reduction in the BST of the CA1 neurons as evidenced by the downward and rightward shift of the I/O curve in the 2VO group. This also accords with our earlier observations (35). It is now well established that peripheral inflammation by microglial activation and excitotoxicity could increase BST (36, 37). Although it is becoming more evident for activation of microglial (38, 39) and excitotoxicity in 2VO, surprisingly we found a reduction in BST of CA1 neurons. The observed decrease in BST may be explained by ischemia reducing calcium-mediated excitatory neurotransmitter release through activation of BK channels (41) and enhancing inhibitory synaptic transmission (40).

Moreover, it has been suggested that the number of hippocampal silent synapses at different time points (1, 4, 12, and 24 weeks) after 2VO model increase by 29.8-55% (41). It is possible to hypothesize that these conditions are likely to occur in our study. In addition, more recently by Haghani research group have been suggested that changes in intrinsic electrophysiological properties of neurons in hepatic encephalopathy induced by bile duct ligation (BDL) strongly depressed BST, which was caused by increases of I_A_, K_Ca_^2+^ and Ca^2+^ currents (5, 12, 42) and MINO restored the depressed BST by improvement of intrinsic electrophysiological properties of neurons (12). However, we found that MINO in both periods (early and late) was failed to recover the depressed BST of the CA1 neurons in 2VO rats, this inconsistency may be due to the different model used (BDL *vs.* 2VO). Therefore, it can be assumed that alteration in intrinsic electrophysiological properties of the hippocampal neurons may not be a factor in 2VO rats for depressed BST. It seems possible that other factors such as high inhibitory synaptic transmission and/or enhancement of silent synapse following 2VO could be significant factors, if not the only one, causing depress BST.

Moreover, we found that the level of PPR for all ISIs is comparable between control, 2VO+V, and 2VO+MINO-E groups. It is well known that the PPR is a good index for measuring release probability; the accumulation of Ca^2+^in the presynaptic terminal from sequential stimulation can lead to an increase in PPR (43). However, with repetitive stimulation by HFS, the PPR tends to be depressed due to the depletion of neurotransmitter stores (26, 44), as observed at the interval of 20 and 25 ms, but 2VO prevented from this suppressive effect. In addition, the late treatment with MINO led to PPR decreased with delivery of HFS similar to that of control group, but early treatment with MINO failed to restore depressive effect of HFS on PPR. Interestingly, we found a high level of PPR before and after HFS in the 2VO+MINO-L group compared to the other groups, the higher level of PPR even after HFS in the MINO-received animals suggested a greater degree of neurotransmitter pool in presynaptic terminal. Shabani *et al*., have been reported same result with treatment of FTY720 in BDL rats. They suggested that FTY720 might decreases the release probability along with an increase in the number of readily releasable pool of neurotransmitter (5). Therefore, an implication of same possibility could be contributed by late treatment of MINO in 2VO rats but implies that the drug effect would be seen only when a late administration was used. Thus, increment of readily releasable neurotransmitter pool could be a direct effect by MINO in 2VO rats. 

Finally, our data indicated impairment in LTP induction of 2VO rats, which was improved only by late treatment of MINO similar to that found in memory assessment. It has previously been observed that different factors exert a powerful effect on LTP disruption after carotid artery occlusion such as, persistent increases in the levels of IL-1β and TNF along with an increase in other inflammatory cytokines like IL-6, MDA, microglial activation (45), synapse loss (41, 46) and decreases in the activity of superoxide dismutase and glutathione peroxidase (47). Notably, the neuroprotective action of MINO administration in CCH was found to be exerted by a decrease of apoptosis and oxidative stress via down-regulation of *iNOS* and up-regulation of *eNOS* (19). In this regard, inhibition of pro-inflammatory cytokine has been suggested as an effective therapeutic strategy in treating hypo-perfused brains (47, 48). It can thus be suggested that all these factors may increase gradually and worsen over time; thus, early treatment with MINO fails to recover memory performance and electrophysiological properties. These may explain the beneficial effects of late treatment of MINO and restoration of behavioral functions. Therefore, a further study with more focus on the profile of inflammatory factors over time is suggested. Since we found an increase in the number of readily releasable neurotransmitter pool by late treatment of MINO, the direct effect of MINO could be another possible explanation for the improvement of LTP and cognition.

## Conclusion

In our study, early treatment with MINO after 2VO did not show long-lasting beneficial effects on cognitive functions and electrophysiological properties. However, the late treatment improved the memory and LTP in 2VO rats. It is possible that MINO improves synaptic plasticity and memory performance in hypo-perfused rats directly by increasing neurotransmitter pool and/or suppressing inflammatory factors.
